# Supplementary material for: A dual fluorescent Plasmodium cynomolgi reporter line reveals in vitro malaria hypnozoite reactivation
Source: Commun Biol. 2020 Jan 3;3:7. doi: 10.1038/s42003-019-0737-3 (PMC6941962; doi:10.1038/s42003-019-0737-3)
Supplement: Supplementary file 1 — Supplementary Information [file 42003_2019_737_MOESM1_ESM.pdf]

a. **b.**

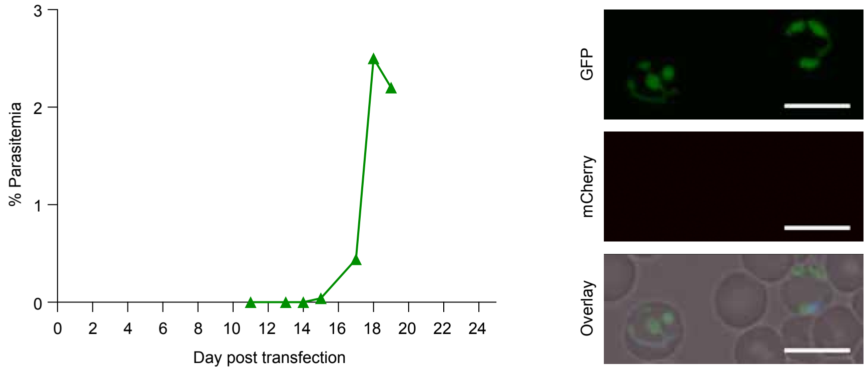

c.

	Feed 1		Feed 2	
	**Average # oocysts per inf. midgut**	**% Infected**	**Average # oocysts per inf. midgut**	**% Infected**
WT	8.9	100	185	100
Transgenic line	4.9	80	181	100

	Feed 2	
	**Total # Spz**	**# Spz/ mosquito**
WT	17 x 10e6	67.5 x 10e3
Transgenic line	11,7 x 10e6	64.6 x 10e3

Supplementary Figure 1. Fitness and characteristics of the transgenic *P. cynomolgi* line. (**a**) Blood stage parasitemia of transfected blood stage parasites in a recipient monkey as assessed by Giemsa stained thin films. (**b**) Live fluorescence images of GFP and (the absence of) mCherry expression in blood stage trophozoites from a monkey infected with the transgenic *P. cynomolgi* line. At the lower panel, an overlay with brightfield and Hoechst 33342 staining the parasite nuclei. Scalebars, 10 µm. (**c**) Average number of oocysts and % of infected mosquitoes counted in 10 mosquito midguts one-week post mosquito feedings (on 2 consecutive days) of blood from a monkey infected with wild type *P. cynomolgi* or the transgenic parasite line. In the lower panel, the number of salivarygland sporozoites is depicted dissected from mosquitoes fed with the wild type (n= 252 mosquitoes) or the transgenic *P. cynomolgi* line (n=181 mosquitoes). Mosquito transmissions were performed at the same time using the same batch of mosquitoes.

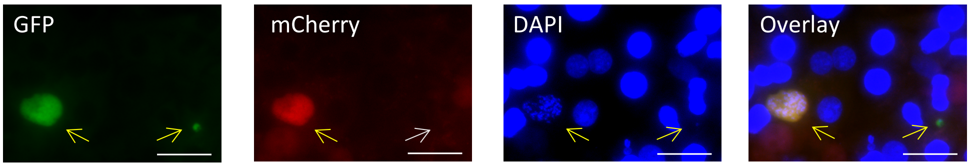

Supplementary Figure 2. GFP and mCherry expression in day 6 liver stage parasites after fixation and DAPI staining. Expression is marked by yellow arrows; absence of mCherry expression is marked by a grey arrow. mCherry is only expressed in the multinucleate parasite and not in the single nucleate parasite (DAPI staining). Scale bars, 25 µm.

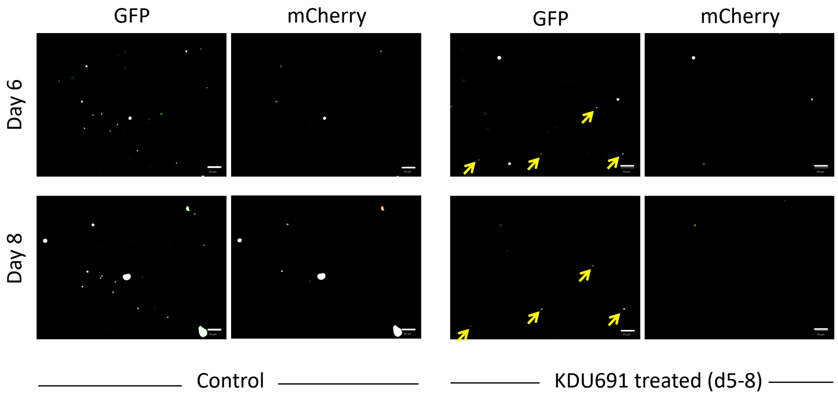

Supplementary Figure 3. Live imaging of control and PI4K inhibitor treated (0.5 µM KDU691, day 5-8) fluorescent parasites at selected time points. The PI4K treatment selectively eliminates liver stage schizonts (GFP and mCherry positive) while hypnozoites (GFP positive, mCherry negative) remain present (yellow arrows)**.** Scale bars, 50 µm.

Supplementary Table 1. Exo-erythrocytic forms (EEF) counted in the same well at days 10 and 15 post sporozoite inoculation.

	Small (GFP+ve/mCherry-ve)			Large (GFP+ve/mCherry+ve)		
	**Day 10**	**Day 15**	**%[1]**	**Day 10**	**Day 15**	**%[1]**
Experiment 1	72	49	68	43	11	26
Experiment 2A[2]	190	61	32	112	22	20
Experiment 2B[2]	170	151	89	78	30	38
Experiment 3	63	48	76	32	10	31
Average EEF @day15			*66*			*29*

[1]*% EEF counts at day 15 versus day 10*

[2]*For experiment 2, the same batch of sporozoites was used to inoculate freshly isolated (2A) or cryopreserved (2B) hepatocytes*
