# Supplementary material for: A dual fluorescent Plasmodium cynomolgi reporter line reveals in vitro malaria hypnozoite reactivation
Source: Commun Biol. 2020 Jan 3;3:7. doi: 10.1038/s42003-019-0737-3 (PMC6941962; doi:10.1038/s42003-019-0737-3)
Supplement: Supplementary file 2 — Description of Additional Supplementary Files [file 42003_2019_737_MOESM2_ESM.docx]

**Description of Additional Supplementary Files**

**Supplementary Data 1.** Cloning scheme and sequences of construct pCyCEN_Lisp2mCherry_hsp70_GFP

**Supplementary Data 2.** Source data for Figure 1b, 3c and 4c.

**Supplementary Movie 1. A transgenic *P. cynomolgi* blood stage merozoite invading a rhesus red blood cell.** A drop of blood obtained from a monkey infected with *P. cynomolgi* transgenic blood stage parasites was viewed under a Leica DMI6000B inverted fluorescence microscope and images were acquired using a DFC365FX camera.

**Supplementary Movie 2. Real-time visualization of exflagellation of a transgenic *P. cynomolgi* gametocyte.** A drop of blood for mosquito feeding obtained from a monkey at day 12 post infection with *P. cynomolgi* transgenic blood stage parasites was viewed under a Leica DMI6000B inverted fluorescence microscope and images were acquired using a DFC365FX camera.

**Supplementary Movie 3. Live fluorescence imaging of transgenic *P. cynomolgi* merosome release.** Live imaging using a Leica DMI6000B inverted fluorescence microscope showing GFP expressing merozoites released from a fully mature *P. cynomolgi* liver stage schizont at day 10 post sporozoite inoculation. Scale bar, 25 µm.

**Supplementary Movie 4.** **Live fluorescence imaging of transgenic *P. cynomolgi* liver stage development in a well from a 96w plate including a hypnozoite reactivation event.** Live images acquired and stitched by an Operetta High Content imager of GFP and mCherry expressing liver stage parasites showing an overview of a well at different days post transgenic *P. cynomolgi* sporozoite infection. The images were compiled to generate a movie revealing the kinetics of *P. cynomolgi* liver stage development over time. The arrow shows a hypnozoite activation event occurring after the first wave of liver stage schizogony has ended.

|  | | | |
| --- | --- | --- | --- |
|  |  |  |  |
