# Supplementary material for: A dual fluorescent Plasmodium cynomolgi reporter line reveals in vitro malaria hypnozoite reactivation
Source: Commun Biol. 2020 Jan 3;3:7. doi: 10.1038/s42003-019-0737-3 (PMC6941962; doi:10.1038/s42003-019-0737-3)
Supplement: Supplementary file 3 — Supplementary Data 1 [file 42003_2019_737_MOESM3_ESM.docx]

| Cloning scheme of construct pCyCEN_Lisp2mCherry_hsp70_GFP  Sequence from plasmid #1 |
| --- |
| **mCherry-2A-Nanoluc** |
|  |
| AGATCTATGGTGAGCAAGGGCGAGGAGGATAACATGGCCATCATCAAGGAGTTCATGCGCTTCAAGGTGCACATGGAGGGCTCCGTGAACGGCCACGAGTTCGAGATCGAGGGCGAGGGCGAGGGCCGCCCCTACGAGGGCACCCAGACCGCCAAGCTGAAGGTGACCAAGGGTGGCCCCCTGCCCTTCGCCTGGGACATCCTGTCCCCTCAGTTCATGTACGGCTCCAAGGCCTACGTGAAGCACCCCGCCGACATCCCCGACTACTTGAAGCTGTCCTTCCCCGAGGGCTTCAAGTGGGAGCGCGTGATGAACTTCGAGGACGGCGGCGTGGTGACCGTGACCCAGGACTCCTCCCTGCAGGACGGCGAGTTCATCTACAAGGTGAAGCTGCGCGGCACCAACTTCCCCTCCGACGGCCCCGTAATGCAGAAGAAGACCATGGGCTGGGAGGCCTCCTCCGAGCGGATGTACCCCGAGGACGGCGCCCTGAAGGGCGAGATCAAGCAGAGGCTGAAGCTGAAGGACGGCGGCCACTACGACGCTGAGGTCAAGACCACCTACAAGGCCAAGAAGCCCGTGCAGCTGCCCGGCGCCTACAACGTCAACATCAAGTTGGACATCACCTCCCACAACGAGGACTACACCATCGTGGAACAGTACGAACGCGCCGAGGGCCGCCACTCCACCGGCGGCATGGACGAGCTGTACAAGGGAGAAGGAAGAGGAAGTTTATTAACATGTGGAGATGTAGAAGAAAATCCAGGACCAGTCTTCACACTCGAAGATTTCGTTGGGGACTGGCGACAGACAGCCGGCTACAACCTGGACCAAGTCCTTGAACAGGGAGGTGTGTCCAGTTTGTTTCAGAATCTCGGGGTGTCCGTAACTCCGATCCAAAGGATTGTCCTGAGCGGTGAAAATGGGCTGAAGATCGACATCCATGTCATCATCCCGTATGAAGGTCTGAGCGGCGACCAAATGGGCCAGATCGAAAAAATTTTTAAGGTGGTGTACCCTGTGGATGATCATCACTTTAAGGTGATCCTGCACTATGGCACACTGGTAATCGACGGGGTTACGCCGAACATGATCGACTATTTCGGACGGCCGTATGAAGGCATCGCCGTGTTCGACGGCAAAAAGATCACTGTAACAGGGACCCTGTGGAACGGCAACAAAATTATCGACGAGCGCCTGATCAACCCCGACGGCTCCCTGCTGTTCCGAGTAACCATCAACGGAGTGACCGGCTGGCGGCTGTGCGAACGCATTCTGGCGTAAGATATC |
|  |
|  |
|  |
| Sequence from plasmid #2 |
| **5LISP2-3LISP2** |
| GGATCCCGGATGTGCGGCGAACCGGTGTGTGTATCAAGGGAAATAAAAAACAAAAACAAAAAACAAAAATAAAAAAACAAAAATGAAATAATAAAAATTAAATTGAATTAAACTGAATTGAGCCAACTGGGCGTAAATTCGAAAAATTTTCTTTTTTTTTAAGCGGAGCTGTTTCCACTTAGTTGTTCATTTGGGGGGAAGCTCTGAGCCACGCTATTTTGCCGGCATGTTTGGCAGTGCACCACCTCCGATGAGCGGAAGAAATGCCAAGTTAAAGACATGCATGAGAATAACGCTTCCATAAGGAAGTGATAAAATAATTCACTTGGGAAAAAAATGAACCACATGGCTTCTTCCCTCGAGGAAGCATCAGCGTTCACCATGCAACGTTTGCGTGTATATTCTTCTTCCCCAAGCGTGCGCCTCGTGACATGTTAGAATTGTTGCAGGCAAAACGAGAGCATCACAGAGGAGACACAATATGTTGAGGAGACATCCAAATGGTAGCGAATCGATCATGCATGGAATAAAGGGCCTCTAAAAAAAGTCATGAAAAATGGCAAAAGGAACAATTCCTCGGGTGACCCACATATTGAGAGCGCCCTTCCACGCTGGTGCAATAAAACAAGTGCAACGATGTGGTCATTTAAAAAATTCAATAATATGAAAGGAAATCTCTTAAGCGATTTGCAAAATTGACGATTTGATAATCAGCAATGGGCTGGGCAGTGTCGAATGACCTTTTTATACATCTTAAATAGGAAAGCTGCTCATCCCCATCCTCCTTTTTTTGCCGAGCGTTAATCGGTTGTATGCCCACTTAGAAACGCTGCATGCACTTCCCCCCGTTGATAATTTTTTTTTTTTTTTGCATAACCTGTTTTATTCTTCCGTGAAAAATGAAAGCATTTGAGGAAATGCTCTTAGTGGTTATTCTAAGTAGGGGTTATCTCATGAGCCCCTAGGGTTCTTCTTTTAAGTGTCTACACAATGCCAATGGCACGTAATCGCTCCATCGTGTAATTGTGTGACTTGAGAGACGGGGGTGGGCAAATGGAACCACATTTGGGAAGCAACGACGTGCTCGATCGTGCGTCAAACGGATCTGCATGCAAAGTTGTTCCAAGTGGAATGGCAAATCTGTGCCAAGTGGAATGGCAAATCTGTGCCATGTGAAATTGTAAAATGAGTACACATTAAGTCGACACAAAAATGTATCATTCAAAACAAAAAAAAAATTTCGAGTCGTCAGTTGACAATTCAGGTTAAGTGAAACTTTACAAATTAAACAACAAAAAATTGCAAGCGGCTAAGTAGCAAAACGGCTGAGCAGCAAAACAGCTGAACAAAACAACTAAAAAAACAACTAAAAAAACACCTGAAAAAACAACTGAAAAAATTGAACAAAACTGAACGGAACATCTTAACAAAACAACTGAGCATCTGCTTAAACTCCATATCTAAACCCTAAATCTTAAACCCTTTTTCGTAAAACTCCTCTCTCCTACTTGCAGATCTGCGGGATATCCCAAGCGGATTAGGCAAACGAAGCAGGGAGACGAAGACGAGTGCGCTCTGACGAACGAAACAACCCCGTCATATTTTTCATGCAACGAAAAGATGGAAAAATACCACACCAAGACAATGACCACCTTGCACGGATATTTGCGACGCGAAAAACGTGTGTTTTAAAACATGGATTAGTATACCATGGGGAAAAACCCATTTCGCATACAAAAGTGCAGGTATCACAGGAGATAATATTTTCTTTCCTTTTCGTAACCACGCATACTGCTCTTTTCATGTGCACTAACCCGCTTTGAGCATTTGAATGGAGCGACCACTGGTGCCCATTTTTTGCATTCTCCTCTTCCAAGTTGCTCGTGGAGTAAAGGCAATTGCACGATTCAGAAATGTGCACGTTTTATCGCATACGTGCATTTTGAGATGAGATTCTTTGAGATGAGATTTTTTGAAGTGAGATTTTTTGAAGTGAGATTTTTTGAGATGAGATTTTTTGAGATGAAATTATTTTTAGATGAGGCACTTATATTTTTTTACCACCCCTGCGGGTAAAGTGTGTCCACGCGCGCCCATTCTGCGTCGGGCATTTACATGGCATGAAATGATTACGCGCGTTTCACCACTTGTGCATGCACCTATATTGTGCACGTGTAGAAGAACGAATTCGTGACGTAAGTTGCGAAGGCAGCAAAGGTAGCAAAATCTGCCAAGAGTGATACGTTGAAAACTGGAAAAAAAAAAAAACTAAGACAAATAAAAAGAAAATCACAAATGATAACACAAGTACAGATCGAGGGGGATTCTTTTTTGAATCTCATATTTAATCGGATTGCAGGGGGTACAGAGTAAATATAAGCAGTCGGCTCAGGTTTGCATACGAGCGACACGGAGAGTGGGTTGCACTGAGACCGTTCAGGTGAAACATATTCTGATTTCACATCAAATGTGCAGCAGTTGAATTTATGGCTAGCCAAATATTTTTCATTTCCTAACAGAGTGGAGGTCTATCTCCCAGTATGCCCATTTTTAAGGCATGTGGTTCTTTGGTTTTGGTACC |
|  |
| Sequence from plasmid #3 |
| **5’Pcyhsp70-hDHFR-2A-GFP-3’Pcyhsp70** |
| GGATCCGGCATCATATAAATATCATCACTGCTTGGGTGAATAGGGGGGATGACTGTGCTTCCTTCTTTATATATATGCCCTTTCTGGAGGGGGTGAAAATGATGTATTGCATGGAGACCATTAAAAAGGGGGGTATATAATATATATAAAAGGAAAAGCTCATTTCCGGCAACCCATTTGGTAAGTTTATGTACACTTTGACGTGGTAAGCAAAAAGGAAAAAAAAAAAGAGAAACAGGTTCATTTCGCAAAAACGCTCATATTTTGCAAAAGCGCTCGTACTGGGTGAGGCTGCCACAAAATTGAGTAAATTCTCTTCTCTGTAAGTGTAGCCAGCAAATAGTACGTGGGGGAGGGGCGGGGCCATTTTTCTTCGCGCATGAGCACCCAAAAAAGCGTACATTAAATTAGCGCCTAGGTTCCGCGGCCGGCTCACGAAAAAATGCACGCATGTAGTTATATATAAATAAATGTACATATATATATGTATGTATATATATACCGCTGCATACACTTGCGATGAGCTGTTGTGCCCGGGGGGAGGGGGCACATCCGGAAGCATATATAAAGAATATGCCGCATATACAATCCTCATACATGTAGCATATATAAACAATAAAGAGGCACGGATTTTTTTTTATATATGCGCATGTACAAGCACCAACTGAAGGGGGACGGGGCCAAATGGCCCCGCGGCCCCTCCTCTTCTTTTCCCACTGGCGGCTTGGAGCACCTGCACAATTTTCGAAAAGTAACGAAATTTAGTCCCCTGATTTTTGAACAAAAAATATGTAAAAAAAATGAAGCTGAAATATTTTATTTTTTTTGCAAAACGGATTTACAAAACATTTTTTATAAACATTTTTTTTTATTTTGCAAAAAAATAAAAAAACAAAACAAAAGTCAAAAAATAATTTATATGATATACATATCATTTTATGTACAGAAAAAATTTATGACCAGTTGAGGCATATTCCCTTTTTTTTTGCGGAATTGTAAAAAATATCGTTTTATTCTCTTTTATTATATTAAAATATAATATAATTGTTTTTTTCATTCCTTTGCGTTTTTTTCCTCGTTGTCCTGTGTTTTCACATTTTGTGGTAAAAAAAAAGAAAAAAGGAAGAAAAGCTTATTTTTTTTTTTTTTTTACGCACGAGAGAACGAAGCACAGTGTATGCTTTTTTTTCCCCCGTGAGTAGCCACTGCTTGTTGTTGCTTCCCATTTTTGCCAAGAAAAATTGGCACAACTGCGCAAGCTGCTGCGGTGTAATTTAAGCACTCTGTACTGTACTCTGTTGAATCTCCCTACCGTTAACGGAGGAGCATTAATAAGAACACAAGCCCAAACCCACCCCCTTTTTTGCGAAATTCGCAACTTTTCTCCCTTTTGAGTTTTTTCAAAAACTAGTATGGTTGGTTCGCTAAACTGCATCGTCGCTGTGTCCCAGAACATGGGCATCGGCAAGAACGGGGACCTGCCCTGGCCACCGCTCAGGAACGAATTTAGATATTTCCAGAGAATGACCACAACCTCTTCAGTAGAAGGTAAGCAGAATCTGGTGATTATGGGTAAAAAAACCTGGTTCTCCATTCCTGAGAAGAATCGACCTTTAAAGGGTAGAATTAATTTAGTTCTCAGCAGAGAACTCAAGGAACCTCCACAAGGAGCTCATTTTCTTTCCAGAAGTCTAGATGATGCCTTAAAACTTACTGAACAACCAGAATTAGCAAATAAAGTAGACATGGTCTGGATAGTTGGTGGCAGTTCTGTTTATAAGGAAGCCATGAATCACCCAGGCCATCTTAAACTATTTGTGACAAGGATCATGCAAGACTTTGAAAGTGACACGTTTTTTCCAGAAATTGATTTGGAGAAATATAAACTTCTGCCAGAATACCCAGGTGTTCTCTCTGATGTCCAGGAGGAGAAAGGCATTAAGTACAAATTTGAAGTATATGAGAAGAATGATGGAGAAGGAAGAGGAAGTTTATTAACATGTGGAGATGTAGAAGAAAATCCAGGACCAAGTAAAGGAGAAGAACTTTTCACTGGAGTTGTCCCAATTCTTGTTGAATTAGATGGTGATGTTAATGGGCACAAATTTTCTGTCAGTGGAGAGGGTGAAGGTGATGCAACATACGGAAAACTTACCCTTAAATTTATTTGCACTACTGGAAAACTACCTGTTCCATGGCCAACACTTGTCACTACTTTCGCGTATGGTCTTCAATGCTTTGCGAGATACCCAGATCATATGAAACAGCATGACTTTTTCAAGAGTGCCATGCCCGAAGGTTATGTACAGGAAAGAACTATATTTTTCAAAGATGACGGGAACTACAAGACACGTGCTGAAGTCAAGTTTGAAGGTGATACCCTTGTTAATAGAATCGAGTTAAAAGGTATTGATTTTAAAGAAGATGGAAACATTCTTGGACACAAATTGGAATACAACTATAACTCACACAATGTATACATCATGGCAGACAAACAAAAGAATGGAATCAAAGTTAACTTCAAAATTAGACACAACATTGAAGATGGAAGCGTTCAACTAGCAGACCATTATCAACAAAATACTCCAATTGGCGATGGCCCTGTCCTTTTACCAGACAACCATTACCTGTCCACACAATCTGCCCTTTCGAAAGATCCCAACGAAAAGAGAGACCACATGGTCCTTCTTGAGTTTGTAACAGCTGCTGGGATTACACATGGCATGGATGAACTATACAAATAAATCGATTCACAAAAATCGCTGCTCAGCACACTGTAGAACGGTATATTATATACTGTCCCAGTTGCGTGAAGAGAGAGGAAAGGGGAATTTATGTATGCTTCCTGCCTCTACATGTGGGGTTTTCCAAGTGCGTTCACCTTTTCCCCCGCGAAGAGAGAGGAGCTTTTTTCCTCCGTGCGCACCTCCCATGTATTGGAAGAGCATACACACACAGTAAAACAACACCATGTGCGTGTGGCCCGTGCGACCCTTTTGAACGCGCGCCTGGTGGGTTTGCATGTGTGTCCCACAAGTGAGTCACTCTCGCCCCCCTCCCAAAAAAAAAAAAAAAAAAAAAAAAAAAAAATAAATAAATAATTCATCGCCACTCATCATTTATCGCGATTCATCATTAACTGTGTTAAGATGTACGTGCGCTCGTTCGTACGTATAGGTCCACGCATGCGTTGTAAATTTATGTAACTTTCTTTTTTTTCTGTAAATTAAGGAGCTATATACATAACATGTGCATGTGAATGCTGATATAAAGGATGCTTAATGAATCGTGCGAATCGTTGCAGATGTATTTTTTTTTTTTTTTAAAAACACTGAAAGAATAGCAACACGAAAAGGAAATTATGTAATCAAAATTGTAGTCACATTGAATTTTTGCATTGGGAAGTGGCGACCCTGGTAGGCCCTGCCCTGTCCATTTGTTATGTGCCGAGTGTGGGGTGGTGAAATCGGTTGTCCCCATTTGGGTAAGATAAGGAAATAGCTTTTCTCAAAGGCATTTCGAAAGGGTGTGTGTTGTATGCACACGTTGGGCGGCCGCGTCGAC |
|  |
