# Supplementary material for: A dual fluorescent Plasmodium cynomolgi reporter line reveals in vitro malaria hypnozoite reactivation
Source: Commun Biol. 2020 Jan 3;3:7. doi: 10.1038/s42003-019-0737-3 (PMC6941962; doi:10.1038/s42003-019-0737-3)
Supplement: Supplementary file 9 — Reporting Summary [file 42003_2019_737_MOESM9_ESM.pdf]

nature research

Corresponding author(s): Clemens Kocken

Last updated by author(s): Dec 5, 2019

Reporting Summary

Nature Research wishes to improve the reproducibility of the work that we publish. This form provides structure for consistency and transparency in reporting. For further information on Nature Research policies, see Authors & Referees and the Editorial Policy Checklist.

Statistics

For all statistical analyses, confirm that the following items are present in the figure legend, table legend, main text, or Methods section.

n/a	Confirmed	
☐	☒	The exact sample size (n) for each experimental group/condition, given as a discrete number and unit of measurement
☐	☒	A statement on whether measurements were taken from distinct samples or whether the same sample was measured repeatedly
☒	☐	The statistical test(s) used AND whether they are one- or two-sided<br>*Only common tests should be described solely by name; describe more complex techniques in the Methods section.*
☒	☐	A description of all covariates tested
☒	☐	A description of any assumptions or corrections, such as tests of normality and adjustment for multiple comparisons
☐	☒	A full description of the statistical parameters including central tendency (e.g. means) or other basic estimates (e.g. regression coefficient) AND variation (e.g. standard deviation) or associated estimates of uncertainty (e.g. confidence intervals)
☒	☐	For null hypothesis testing, the test statistic (e.g. F, t, r) with confidence intervals, effect sizes, degrees of freedom and P value noted<br>*Give P values as exact values whenever suitable.*
☒	☐	For Bayesian analysis, information on the choice of priors and Markov chain Monte Carlo settings
☒	☐	For hierarchical and complex designs, identification of the appropriate level for tests and full reporting of outcomes
☒	☐	Estimates of effect sizes (e.g. Cohen's d, Pearson's r), indicating how they were calculated

Our web collection on statistics for biologists contains articles on many of the points above.

Software and code

Policy information about availability of computer code

Data collection	Fluorescent parasite images obtained with the Leica DMI6000B microscope were acquired using Las X software from Leica. Fluorescent images for tracking parasites were acquired using an Operetta High Content imager with Harmony software 4.1.
Data analysis	Images acquired with the Operetta High Content Imager were analyzed using Columbus 2.8.2 software (Perkin-Elmer). Analyses were performed with Microsoft Excel for Mac version 16.16.11. Analyses and graphs were made using GraphPad Prism 7 software.

For manuscripts utilizing custom algorithms or software that are central to the research but not yet described in published literature, software must be made available to editors/reviewers. We strongly encourage code deposition in a community repository (e.g. GitHub). See the Nature Research guidelines for submitting code & software for further information.

Data

Policy information about availability of data

All manuscripts must include a data availability statement. This statement should provide the following information, where applicable:

- Accession codes, unique identifiers, or web links for publicly available datasets
- A list of figures that have associated raw data
- A description of any restrictions on data availability

The data and rescources that support the findings of this study are available from the corresponding author upon reasonable request. The transfection construct pCyCEN_Lisp2mCherry_hsp70_GFP can be requested through Addgene, ID 137169.

Field-specific reporting

Please select the one below that is the best fit for your research. If you are not sure, read the appropriate sections before making your selection.

☒ Life sciences ☐ Behavioural & social sciences ☐ Ecological, evolutionary & environmental sciences

For a reference copy of the document with all sections, see nature.com/documents/nr-reporting-summary-flat.pdf

Life sciences study design

All studies must disclose on these points even when the disclosure is negative.

Sample size	Sample sizes were estimated empirically, based on other studies in the field.
Data exclusions	Parasites that express mCherry prior to day 10 were excluded from analyses for hypnozoite activation.
Replication	Five independent infections were performed to examine hypnozoite activation events. These infections were initiated with sporozoites from 5 different infections of 5 different donor monkeys using different batches of mosquitoes.
Randomization	Randomization was not relevant to this study. There were no experimental groups involved.
Blinding	Blinding was not relevant in this work.

Reporting for specific materials, systems and methods

We require information from authors about some types of materials, experimental systems and methods used in many studies. Here, indicate whether each material, system or method listed is relevant to your study. If you are not sure if a list item applies to your research, read the appropriate section before selecting a response.

Materials & experimental systems

n/a	Involved in the study	
☐	☒	Antibodies
☒	☐	Eukaryotic cell lines
☒	☐	Palaeontology
☐	☒	Animals and other organisms
☒	☐	Human research participants
☒	☐	Clinical data

Methods

n/a	Involved in the study	
☒	☐	ChIP-seq
☒	☐	Flow cytometry
☒	☐	MRI-based neuroimaging

Antibodies

Antibodies used	Rabbit anti-P. cynomolgi HSP70.1 polyclonal antibody; used at 1/10,000 dilution.<br><br>Alexa Fluor 647-conjugated Goat anti-Rabbit IgG (H+L) Thermo Fisher Scientific Cat#A21245; dilution 1/1000.
Validation	Rabbit anti-P. cynomolgi HSP70.1 polyclonal antibody has been described in ref. 31.<br>For the Alexa Fluor 647conjugated Goat anti-Rabbit IgG (H+L) Thermo Fisher Scientific Cat#A21245 information is available from the manufacturer: https://www.thermofisher.com/antibody/product/Goat-anti-Rabbit-IgG-H-L-Highly-Cross-Adsorbed-Secondary-Antibody-Polyclonal/A-21245

Animals and other organisms

Policy information about studies involving animals; ARRIVE guidelines recommended for reporting animal research

Laboratory animals	Macaca mulatta, either gender, age 4-16 years, Indian origin
Wild animals	The study did not involve any animals from the wild.
Field-collected samples	The study did not involve samples collected from the field.
Ethics oversight	The research protocol was approved by the central committee for animal experiments (CCD license number AVD5020020172664) and the subprotocol was approved by the local independent ethical committee constituted conform Dutch

law (BPRC Dier Experimenten Commissie, DEC; agreement number #708 and #007C) prior to the start of the experiments. All experiments were performed according to Dutch and European laws. The Council of the Association for Assessment and Accreditation of Laboratory Animal Care (AAALAC International) has awarded BPRC full accreditation. Thus, BPRC is fully compliant with the international demands on animal studies and welfare as set forth by the European Council Directive 2010/63/EU, and Convention ETS 123, including the revised Appendix A as well as the 'Standard for humane care and use of Laboratory Animals by Foreign institutions' identification number A5539-01, provided by the Department of Health and Human Services of the United States of America's National Institutes of Health (NIH) and Dutch implementing legislation.

Note that full information on the approval of the study protocol must also be provided in the manuscript.
